# Supplementary material for: Model-based evaluation of alternative reactive class closure strategies against COVID-19
Source: Nat Commun. 2022 Jan 14;13:322. doi: 10.1038/s41467-021-27939-5 (PMC8760266; doi:10.1038/s41467-021-27939-5)
Supplement: Supplementary file 1 — Supplementary Information [file 41467_2021_27939_MOESM1_ESM.pdf]

# Supplementary Information

## Model-based evaluation of alternative reactive class closure strategies against COVID-19

Quan-Hui Liu[1]*, Juanjuan Zhang[2,3,4]*, Cheng Peng[2], Maria Litvinova[5], Shudong Huang[1], Piero Poletti[6], Filippo Trentini[7], Giorgio Guzzetta[6], Valentina Marziano[6], Tao Zhou[8], Cecile Viboud[9], Ana I. Bento[5], Jiancheng Lv[1], Alessandro Vespignani[10,†], Stefano Merler[6,†], Hongjie Yu[2,3,4,†,#], Marco Ajelli[11,†,#]

**Author Affiliations**

1. College of Computer Science, Sichuan University, Chengdu, China
2. School of Public Health, Fudan University, Key Laboratory of Public Health Safety, Ministry of Education, Shanghai, China
3. Shanghai Institute of Infectious Disease and Biosecurity, Fudan University, Shanghai, China
4. Department of Infectious Diseases, Huashan Hospital, Fudan University, Shanghai, China
5. Department of Epidemiology and Biostatistics, Indiana University School of Public Health, Bloomington, IN, USA
6. Center for Health Emergencies, Bruno Kessler Foundation, Trento, Italy
7. Dondena Centre for Research on Social Dynamics and Public Policy, Bocconi University, Milan, Italy
8. Big Data Research Center, University of Electronic Science and Technology of China, Chengdu, China
9. Division of International Epidemiology and Population Studies, Fogarty International Center, National Institutes of Health, Bethesda, MD, USA
10. Laboratory for the Modeling of Biological and Socio-technical Systems, Northeastern University, Boston, MA, USA
11. Laboratory for Computational Epidemiology and Public Health, Department of Epidemiology and Biostatistics, Indiana University School of Public Health, Bloomington, IN, USA

*These authors contributed equally. [†]These authors are joint senior authors. [#]Corresponding authors

**This PDF file includes:**

Supplementary Methods
Supplementary Figures 1-18
Supplementary Tables 1-3
Supplementary References

## Contents

*Supplementary Methods* .......... 3
1 SARS-CoV-2 transmission model .......... 3
1.1 Synthetic population .......... 3
1.2 Transmission model .......... 3
1.3 Reproduction number .......... 7
1.4 Simulated intervention strategies .......... 7
1.5 Model parametrization .......... 11
2 Reactive class-closure strategy based on syndromic surveillance .......... 13
2.1 Additional results for the baseline analysis .......... 13
2.2 Daily number of imported infections .......... 15
2.3 Incubation period .......... 16
2.4 Homogeneous susceptibility to infection by age .......... 17
2.5 Infectiousness of asymptomatic vs. symptomatic individuals .......... 18
2.6 Initial fraction of immune population .......... 19
3 Reactive school-closure strategy based on syndromic surveillance .......... 21
4 Reactive class-closure strategy based on rapid antigen screening .......... 22
4.1 Additional results for the baseline analysis .......... 22
4.2 Sensitivity of antigen test .......... 25
4.3 Antigen screening frequency .......... 26
4.4 Initial fraction of immune population .......... 29
*Supplementary References* .......... 30

*Supplementary Methods*

# 1 SARS-CoV-2 transmission model

## 1.1 Synthetic population

We adopted the algorithm used for modeling contact patterns in European populations [1] to build a synthetic population of about 500,000 individuals. The modelled population mimics the sociodemographic structure of the actual Italian population in terms of age structure, household size distribution and within-household age composition, school attendance rates, school size distribution, class size distribution, and the number of teachers per students.

Each single individual is explicitly represented in the model as an agent. The network of contacts between individuals can be described by three different layers accounting for contacts between household members, between schoolmates (and classmates within each school), and all other contacts occurring in the general community (i.e., contacts related to leisure activities, use of transportation means, and contacts occurring among work colleagues, etc.). Households are defined as $n_h$ disconnected components (i.e., the total number of households ) grouping a number of individuals sampled from the actual Italian household size distribution [2]. In the model, individuals' age is determined by an algorithm tailored to reproduce realistic age-gaps between household members for a given household size and to match the actual Italian age distribution [1, 2].

Similar to the household layer, schools of different types (primary, middle, and high schools) are defined as $n_s$ (i.e., the total number of schools) disconnected components with size sampled from the Italian distribution of school size. Individuals are assigned to different schools taking into account age-specific school enrollment rates and the teacher-to-student ratio associated to each school type, as reported in the official statistics. Briefly, students are randomly assigned to one single class and one single school using a heuristic approach that allows us to mirror the average, minimum, and maximum class size reported in the official records, and the age composition within classes. Teachers of each school are randomly sampled from the individuals of the synthetic population based on age distribution of teachers in Italy.

The general community layer is represented by one single fully connected component consisting of all individuals in the population and aims at representing the network of all contacts occurring outside households and schools.

## 1.2 Transmission model

In the synthetic population, SARS-CoV-2 transmission is simulated using a discrete-time stochastic Markov process. We consider that each individual can be characterized by one

of five mutually exclusive epidemiological states (corresponding to the states of the Markov process): susceptible (i.e., individuals who may acquire infection after exposure to SARS-CoV-2 infected individuals), infectious pre-symptomatic (i.e., individuals who are not showing any clinical sign or symptom but that are able to transmit the infection and will develop symptoms in the future), infectious symptomatic (i.e., individuals who developed symptoms and are able to transmit the infection), infectious asymptomatic (i.e., individuals who are able to transmit the infection, but that will not developed symptoms), and removed (i.e., individuals who recovered from the infection gaining immunity against re-infection).

In the model, SAR-CoV-2 transmission occurs upon contacts between susceptible and infectious individuals taking place in one of four transmission settings (household $h$, school $s$, class $c$ within the school, and the general community $r$). At each time step $t$ (corresponding to one day), the probability that a susceptible individual $i$ is infected through a contact with an infectious individual $j$ in setting $l$ is modeled as:

$$\mathrm{p}_{[\mathrm{j}\to\mathrm{i}]}(\mathrm{t}) = \beta_l \delta(a_i)\chi(\mathrm{s_j})/n_{l(j)} \qquad 1.1$$

where:

- $\beta_l$ is the daily per contact transmission rate, shaping the risk of infection due to interactions with an infectious individual ($\mathrm{day}^{-1}$) in setting $l$, $l \in \{h, s, c, r\}$.
- $n_{l(j)}$ is the number individuals in the household ($l = h$), school ($l = s$), class ($l = c$) where individual $j$ belongs to. Since all individuals are potentially in contact in the community, for $l$=r, $n_{l(j)}$ corresponds to the total number of individuals in the population.
- $a_i$ is the age of individual $i$.
- $\delta(a_i)$ is relative susceptibility to SARS-CoV-2 infection at age $a_i$ (Supplementary Table 1).
- $\mathrm{s_j}$ is dummy variably identifying whether individual $j$ is symptomatic/pre-symptomatic or asymptomatic. To determine whether the infectious individual of age $a_i$ will develop clinical symptoms or not, we draw a random sample from a Bernoulli distribution with probability $s(a_i)$ at the time when $i$ acquires the infection (Supplementary Table 2).
- $\chi(\mathrm{s_j})$ is the relative infectiousness of asymptomatic to symptomatic individuals.

After the latent period, infected individuals are infectious before developing symptoms (pre-symptomatic stage, which is assumed to last 2 days on average[3,4]). The incubation defines the time interval between acquiring the infection and the development of symptoms; in the model, the incubation period is sampled from a Gamma distribution of mean 6.3 days and SD 4.3[3]. The incubation period is key in our analysis, as the syndromic surveillance is based on the detection of symptomatic individuals (either at school or in the

community). The time from the day of symptom onset to recovery is assumed to follow an exponential distribution, whose mean value (M) is estimated so that the generation time inferred from model simulations matches empirical estimates of 6.6 days[5,7].

According to the estimates reported in previous studies, we assume that the infectiousness of pre-symptomatic, symptomatic, and asymptomatic individuals is the same, $\chi = 1$ [3]. However, we performed a sensitivity analysis to explore how model outcomes change when asymptomatic individuals are assumed to be half as infectious compared to symptomatic and pre-symptomatic cases (i.e., $\chi$=0.5).

**Supplementary Table 1**. Summary of model parameters.

| Param | Description | Value (or range) / Procedure | Sensitivity analysis | Reference |
|---|---|---|---|---|
| **Natural history** | | | | |
| - | Incubation period (days) | Gamma distribution with mean=6.3 days, sd=4.3 | Gamma distribution (mean=5.2, sd=4.3) | Hu et al. [3] |
| $T_g$ | Generation time (days) | 6.6 days [95%IQR: 0.7-19.0] | - | Cereda et al. [5] Lavezzo et al. [7] |
| $R$ | Reproduction number (when schools are open) | 1.3, 1.5, 1.7, 1.9 | - | ISS [8] |
| - | Initial immunity | 5% | 10%, 15%, 20% | Marziano et al. [9] |
| **Parameters related to the infection transmission process** | | | | |
| $\beta_h$ | Transmission rate in household | Set to obtain a household secondary attack rate of 51.5% according to Poletti et al. [10] | - | Derived |
| $\beta_r$ | Transmission rate in the community | Set to obtain the desired value of $R$ when schools are closed | - | Derived |
| $\beta_s$ | Transmission rate between schoolmates | Set to obtain the desired value of $R$ when schools are open | - | Derived |
| $\beta_c$ | Transmission rate between classmates | $\beta_c = \beta_s * \frac{N_c}{N_s}$ where $N_c = 6.3$ (mean daily number of contacts by students with classmates) and $N_s = 1.5$ (mean daily number of contacts by students with schoolmates) according to Litvinova et al. [11] | - | Derived |
| $\delta_a$ | Susceptibility to infection by age $a$ | $\delta_a = 0.56, a < 20$; $\delta_a = 1,\ a \geq 20$ | $\delta_a$=1 for all ages | Viner et al. [12] |
| $\chi$ | Transmissibility of asymptomatic relative to symptomatic individuals | 100% | 50%, 25% | Hu et al. [3] |
| **Reactive school closure** | | | | |
| | Number of days of class closure | 14 days | - | ISS [13] |
| **PCR test** | | | | |

| | | | | |
|---|---|---|---|---|
| $\alpha_s$ | Probability of being tested if symptomatic for students | 95% | 80% | Assumed |
| $\alpha_p$ | Probability of being tested if symptomatic for non-students (passive surveillance) | 45% | 20%, 70% | Assumed |
| $\phi_p$ | Sensitivity of PCR test given the delay between symptom onset and test | Variable over time (values taken from the reference) | - | Kucirka et al. [14] |
| $T_{ST}$ | Delay from symptom onset to sample collection | 2 | 1, 4 | ISS [15] |
| $T_{TR}$ | Delay from sample collection to PCR results | 2 | 1, 4 | ISS [15] |
| **Rapid test** | | | | |
| $n_0$ | Daily number of positive individuals in the population to trigger the student screening strategy | 1 | 0, 5 | Assumed |
| T | All students are tested every T days, if the number of positive individuals in the population is $\geq n_0$ | 3, 7, 14 | - | Assumed |
| $\alpha_r$ | Probability of complying with the screening | 100% | 50%, 75%, 90% | Assumed |
| $\phi_a$ | Sensitivity of antigen test# | 0.69 | Variable over time* | Meta-analysis of sensitivity (Supplementary Fig. 1) |
| **Routine quarantine** | | | | |
| q | Probability of quarantine the household if one member tested as positive | 0.95 | - | Assumed |

# The result of the test is available on the same day the test is performed.

* Same temporal trend of used for the PCR test.

**Supplementary Table 2.** Age-specific parameters regulating COVID-19 disease burden.

| Age group (years) | Probability that an infected individual will develop respiratory symptoms and/or fever [10] (mean, 95% CI) | Probability that an infected individual will require hospitalization [16] (mean, 95% CI) | Probability that an infected individual will develop critical illness [10] (mean, 95% CI) | Infection fatality risk [17] (mean, 95% CI) |
|---|---|---|---|---|
| **0-14** | 18.1% (13.9-22.9%) | 2.5% (1.0-5.1%) | 0.0% (0.0-1.3%) | 0.0% (0.0-1.94%) |
| **15-19** | 18.1% (13.9-22.9%) | 7.4% (0.9-24.3%) | 0.0% (0.0-12.8%) | |
| **20-39** | 22.4% (18.9-26.2%) | 5.3% (3.5 -7.5%) | 0.4% (0.0-1.4%) | 0.0% (0.0-0.9%) |
| **40-49** | 30.5% (27.7-33.5%) | 13.0% (11.0-15.2%) | 0.9% (0.4-1.7%) | |
| **50-59** | 30.5% (27.7-33.5%) | | | 0.4% (0.0-2.0%) |
| **60-69** | 35.5% | 17.2% | 2.6% | 0.9% |

| | | | | |
|---|---|---|---|---|
| | (32.2-38.8%) | (14.0-20.8%) | (1.4-4.5%) | (0.1-3.2%) |
| **70-79** | 35.5% (32.2-38.8%) | 27.5% (22.8-32.6%) | 7.2% (4.6-10.5%) | 5.6% (2.5-10.7%) |
| **≥ 80** | 64.6% (56.6-72.0%) | 43.0% (35.2-51.1%) | 18.4% (12.7-25.3%) | 8.1% (3.3-16.1%) |

## 1.3 Reproduction number

A fundamental epidemiological parameter measuring the potential spread of infection is represented by the reproduction number $R$, which is defined as the number of secondary cases generated by a typical infector in a partially immune population. In our simulations, we explore scenarios of $R$ ranging from1.3 to 1.9, therefore encompassing estimates of $R$ associated to SARS-CoV-2 transmission dynamics observed in fall of 2020 in Italy.

We use a well-known relation [18] between the reproduction number, the distribution of the generation time, and the exponential epidemic growth rate $r$ to estimate the reproduction number in model simulation:

$$R = \frac{\boldsymbol{r}}{\sum_{i=1}^{n} \boldsymbol{y_i}(\boldsymbol{e}^{-\boldsymbol{r}\boldsymbol{a}_{i-1}} - \boldsymbol{e}^{-\boldsymbol{r}\boldsymbol{a}_i})/(\boldsymbol{a}_i - \boldsymbol{a}_{i-1})}$$

where $a_0$, $a_1$,…, $a_n$, are the category bounds of the histogram of the generation time, $y_1$, $y_2$, …, $y_n$ are the corresponding relative frequencies where the observed generation time are within these bounds, and $r$ is the exponential growth rate derived from the analysis of the number of new cases over time in the simulated epidemics.

## 1.4 Simulated intervention strategies

### Reactive class closure based on syndromic surveillance

In this study, we explicitly model the case isolation, quarantine of contacts, and reactive class-closure policy as implemented in Italy since mid-September 2020. The strategy is based on identification of infections among symptomatic individuals in the population using reverse transcription polymerase chain reaction (RT-PCR) testing. The simulated strategy entails the following steps:

- If an individual shows respiratory symptoms and/or fever, they are tested with RT-PCR with probability $\alpha_p = 0.45$ if they are a non-student population or $\alpha_s = 0.95$ if they are a student. The larger probability of being tested used for students stems from the routine temperature screening adopted in most Italian schools at the time. Other values of $\alpha_s$ and $\alpha_p$ are explored in the sensitivity analyses.
- While waiting for sample collection ($T_{ST}$ days after symptom onset, see Supplementary Table 1) and test result ($T_{TR}$ days after sample collection, see Supplementary Table 1),

the symptomatic individual is precautionarily quarantined in their place of residence, while the other members of their households are allowed to continue their normal activities.

- If the result of the test is negative, the tested individual goes back to their normal activities.
- If the result of the test is positive, then:
    - If they are a student, teaching activities for their class are suspended (while the other classes of their school remain open). Note that although their class is closed and thus their classmates cannot attend school, they are not quarantined and thus could potentially infect their household members and other individuals in the general population (should they be infectious).
    - Regardless of whether the positive individual is a student or not, they are isolated at home for 14 days starting with the date of laboratory confirmation.
    - Considering a compliance rate q=95%, the household members of a positive individual are tested with RT-PCR and are precautionarily quarantined at home for 2 weeks starting from the date of laboratory confirmation.
        - If any of their household members is confirmed to be positive with RT-PCR:
            - They remain in isolation at home for 14 days (starting from the date of laboratory confirmation).
            - Moreover, if they are a student, the teaching activities in their class are suspended (starting from the date of confirmation), while the other classes in their school remain open. Note that although their class is closed and thus their classmates cannot attend school, they are not quarantined and thus could potentially infect their household members and other individuals in the general population (should they be infectious).

Symptomatic non-student individuals have 45% probability of being tested. This parameter is set so that in the model simulations the case ascertainment ratio for any symptomatic individual in the population is 31%, matching the value estimated for Italy[19].

It is important to stress that other (not individually targeted) social distancing measures were implemented in Italy (e.g., closure of gyms, limited size of gatherings, use of masks). All those interventions are taken into account in the model as an ensemble, by considering different values of the reproduction number (as estimated in the absence of the test-based interventions mentioned above).

**Reactive class closure based on rapid antigen screening**

Antigen-based tests are commonly used in the diagnosis of respiratory pathogens, including influenza viruses and respiratory syncytial virus. The U.S. Food and Drug Administration (FDA) has granted emergency use authorization (EUA) for antigen tests to identify SARS-CoV-2 [20]. Antigen tests are immunoassays that detect the presence of a specific viral antigen, which implies current viral infection. Most of the currently authorized tests can be used at the point of care, return results in approximately 15 minutes, and are relatively inexpensive compared to RT-PCR tests. Antigen tests for SARS-CoV-2 are generally less sensitive than RT-PCR for detecting the presence of viral nucleic acid.

To estimate the sensitivity and specificity of antigen tests for SARS-CoV-2 through a meta-analysis, we conducted a literature review in PubMed and Web of Science. Seven studies reporting the sensitivity and specificity of nine rapid SARS-CoV-2 antigen-detection tests were included: Chaimayo 2020 [21]; Diao 2020 [22]; Lambert-Niclot 2020 [23]; Mertens 2020 [24]; Nalumansi 2020 [25]; Porte 2020 [26]; Weitzel 2020 [27]. The study by Weitzel et al. compared three antigen tests. The original sensitivity and specificity estimates extracted from the identified studies are summarized in Supplementary Table 3. We estimated the overall sensitivity and specificity through our meta-analysis. A random-effect model was used to estimate the pooled sensitivity and specificity, which resulted to be 69% (95%CI: 41%-97%) and 99% (95%CI: 97%-100%), respectively (Supplementary Figure 1).

To the best of our knowledge, no data is available about the sensitivity of antigen tests given the delay between the date of infection and the date of test. As such, we keep it fixed (at 69%) in the baseline analysis and perform a sensitivity analysis (Supplementary Material, Sec. 4.2) where we assume that the sensitivity of the antigen test follows the same temporal trend of the RT-PCR test, although with a lower absolute value.

**Supplementary Table 3.** Summary of the original sensitivity and specificity used in the meta-analysis.

| Study | Sensitivity (mean, 95% CI) | Specificity (mean, 95% CI) |
|---|---|---|
| Chaimayo 2020 | 98% (91%, 100%) | 99% (97%, 100%) |
| Diao 2020 | 76% (69%, 81%) | 100% (91%, 100%) |
| Lambert-Niclot 2020 | 50% (40%, 61%) | 100% (92%, 100%) |
| Mertens 2020* | 58% | 100% |
| Nalumansi 2020 | 70 % (60%, 79%) | 92% (87%, 96%) |
| Porte 2020 | 94% (87%, 97%) | 100% (92%, 100%) |
| Weitzel 2020 [A] | 62% (51%, 72%) | 100% (89%, 100%) |
| Weitzel 2020 [B] | 17% (10%, 17%) | 100% (89%, 100%) |
| Weitzel 2020 [C] | 85 % (76%, 91%) | 100% (89%, 100%) |

Note: *95% CI of the sensitivity and specificity is not available in this study.

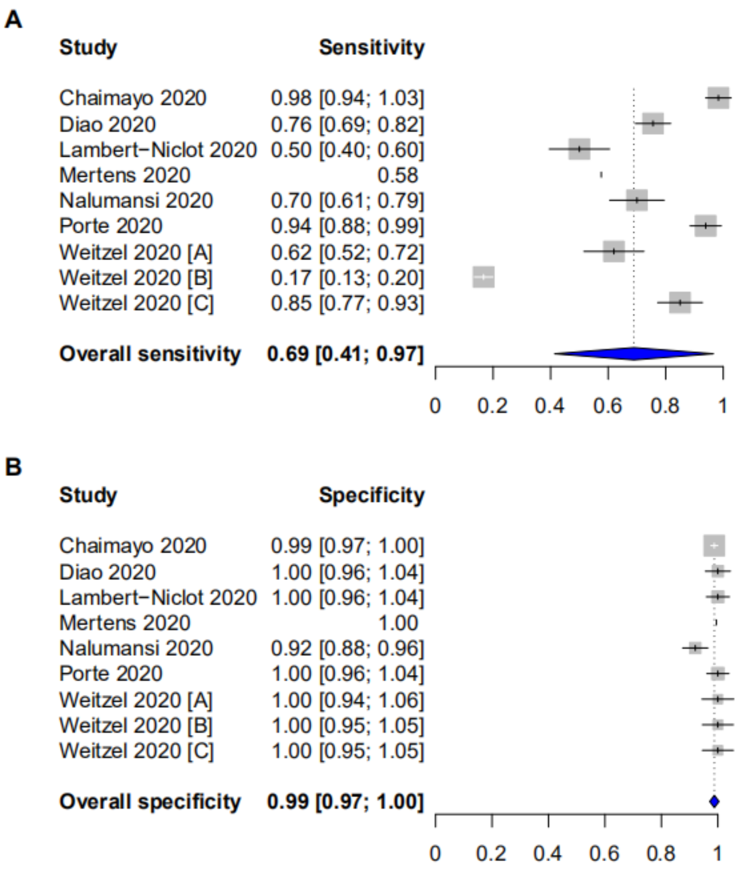


**Supplementary Fig. 1. Results of the meta-analysis about the sensitivity and specificity of antigen tests.** **A** Sensitivity of antigen tests. The boxplots show the quantiles 0.025, 0.25, 0.5, 0.75, and 0.975 of the distribution. **B** As A, but for the specificity of antigen tests.

Taking the advantage of the timeliness and cost of antigen tests, we propose a reactive class closure based on the periodic antigen screening on all students irrespectively of their symptoms or clinical signs. Note that the regular testing via PCR of symptomatic individuals in the population is considered to remain in place. According to this strategy, as soon as a student results positive to either a RT-PCR test performed within the syndromic surveillance or to an antigen test regularly applied to all students, the closure of the class of the infected student is imposed, following the same procedure already in place for reactive class closure (see previous section).

In our analysis, we explored three different frequencies for antigen screening: every 3 days, every 7 days, and every 14 days. As rapid antigen tests give very timely results, we assume that laboratory diagnosis from these tests is obtained on the same day of the sample collection.

## 1.5 Model parametrization

**Initialization.** We initialized the population to reflect the epidemiological conditions characterizing Italy in September 2020. We therefore assume that, at the beginning of our simulations, 5% of the Italian population is immune against SARS-CoV-2 infection. This percentage represents the proportion of the Italian population who experienced the SARS-CoV-2 infection during the first-wave of COVID-19, in spring 2020 [19]. The same immunity level was assumed across different age groups. Alternative values of the initial fraction of immune population are explored as sensitivity analysis as well as to simulate an epidemiological situation closer to that of spring 2021. We also performed a sensitivity analysis where we consider immunity to be age dependent. Specifically, we considered that 11.2% of individuals aged 18 years or less are immune and 22.4% of the rest of the population is immune (so that the total fraction of immune population is 20%). Simulations consider that newly infectious individuals are imported from outside the study area on a continuous basis. Specifically, at each time step of the simulation (1 day), we sample from a Poisson distribution of mean 1.34, based on the estimates reported by the national surveillance system (i.e., 0.027 imported cases per day per 10,000 individuals). We have conducted a sensitivity analysis where we consider the mean daily imported infectious individuals to be 0.25 and 4 times the value in the baseline. The sensitivity analysis shows little/no impact of the daily number of imported cases to the outcome of interest in this study (see Sec. 2.2).

**Model calibration.** In Italy, after the first COVID-19 case was identified in February 2020, all teaching activities were completely suspended. All schools in the entire country remained closed until September 2020. In order to be consistent with epidemiological evidences characterizing the first epidemic wave in Italy, model parameters shaping the transmission potential of SARS-CoV-2 (Equation 1.1) and the relative contribution of households in the transmission of the infection were assumed in such a way to reproduce a reproduction number ($R$) of 1.1 [8] and a household secondary attack rate (hSAR) of 51.5% [10]. In our simulation, we consider that after school reopening, $R$ increases to values in the range of 1.3 to 1.9, as estimated in Italian regions [8].

As direct quantitative estimates of the contribution of school (or school-related) activities to the increase in the overall transmissibility are unavailable, we consider three scenarios. In the first one we assumed that the increased transmission observed in Italy during September can be entirely ascribed to transmission in schools (F100). The other two scenarios (F50 and F25) account for the increase in the number of contacts in the general community related to the reactivation of teaching activities, such as contacts made on transportation means, extracurricular activities, etc.

In scenario F100, we kept the transmission rates in household and the community as estimated for the summer period and set the transmission in school to obtain the target value of the reproduction number, which corresponds to attributing 100% of the observed increase of the reproduction number in September/October to school transmission. We run the calibrated model and we estimated the fraction of infections generated in schools, which we denote as $F_S$. In scenarios F50 and F25, we assume that the fraction of infections is $0.5*F_S$ and $0.25*F_S$, respectively. To do so, we fixed the transmission rate in household as in F100, and we re-estimate the transmission rates in school and in the community to obtain the target value of the reproduction number and of the fraction of infections occurring in schools. The total school contribution to infections estimated by the model for the three scenarios (F25, F50, and F100) and different values of the reproduction number is shown in Supplementary Fig. 2.

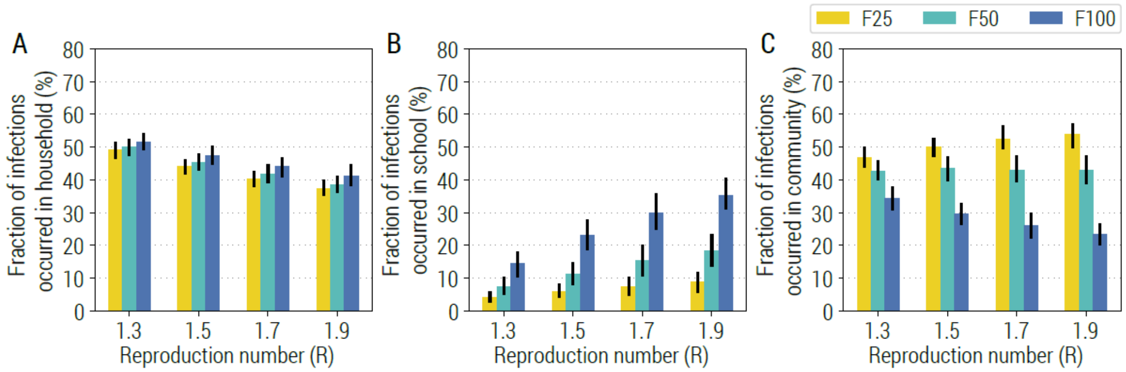


**Supplementary Fig. 2. Estimated fraction of infections by setting. A** Fraction of infections linked to household transmission for different values of *R* and in the three school transmission contribution scenarios. The bar corresponds to the mean value, while the vertical line represents 95% quantile intervals. **B** As A, but for school. **C** As A, but for community.

## 2 Reactive class-closure strategy based on syndromic surveillance

### 2.1 Additional results for the baseline analysis

In addition to the primary results related to COVID-19-related burden reported in the main text (Fig. 1A and 1B), here we show other metrics (Supplementary Fig. 3). Specifically, we estimate that the relative burden of the number of symptomatic infections, hospitalized patients, and patients developing critical illness are similar to those estimated for infections and deaths.

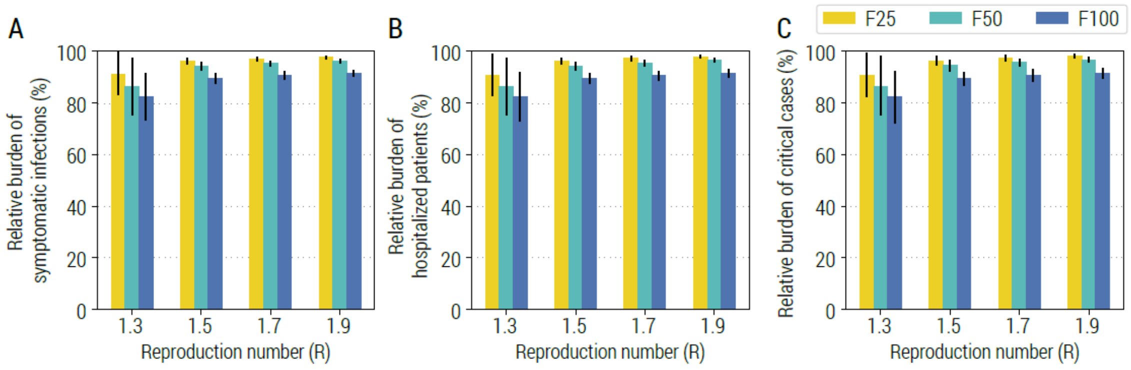


**Supplementary Fig. 3. Impact of the reactive class-closure policy based on syndromic surveillance. A** Relative burden of the cumulative number of symptomatic infections after one year as a function of the reproduction number and for different scenarios about school transmission contribution. The bars correspond to the mean value, while the vertical lines represent 95% quantile intervals; colors refer to the three scenarios F25, F50, F100. Parameters are as the baseline values reported in Supplementary Table 1 and 2. **B** As A, but for the number of hospitalized patients. **C** As A, but for the number of critical cases.

In the main text, we showed the results of four sensitivity analyses considering changes in parameters regulating the implementation of the reactive class-closure strategy. In particular, we varied (i) the probability to test a symptomatic student at school, (ii) the probability to test a symptomatic (non-student) individual in the community, (iii) the time from symptom onset to sample collection, and (iv) the time from sample collection to laboratory diagnosis. Supplementary Fig. 4 shows the impact of these parameters on the proportion of infectious students in each open class of a school other than the class triggering the class closure. Under all the analyzed scenarios, for R>1.5, we estimate the median of this proportion to be around 5% with peak values exceeding 20%. This finding highlights that even quicker or more intense syndromic surveillance would not be able to identify infected students readily enough to prevent widespread school transmission before class closures are triggered.

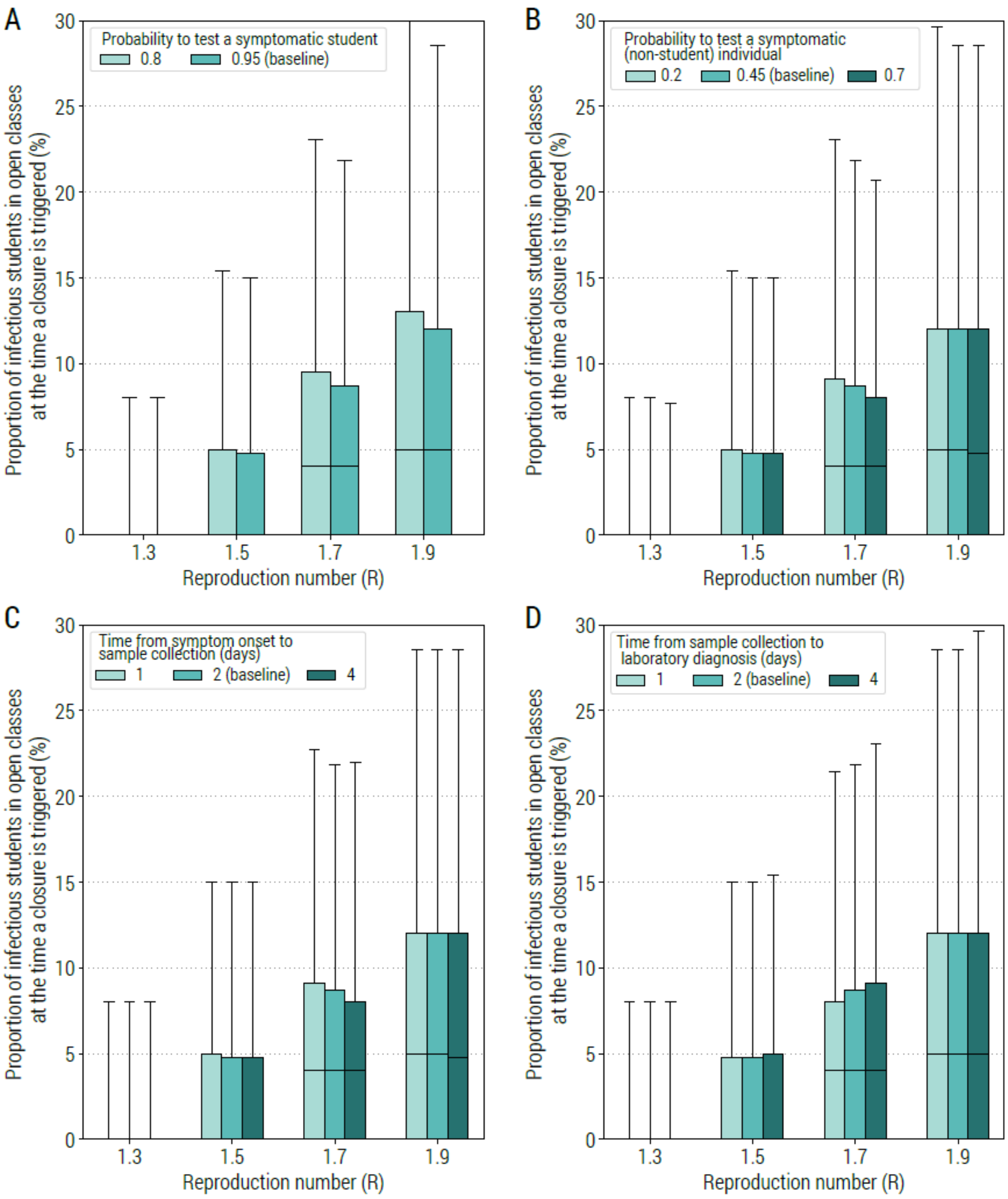


**Supplementary Fig. 4. Sensitivity of the class-closure strategy based on syndromic surveillance to changes in parameters regulating its implementation. A** Proportion of infectious students in all the open classes of the school (i.e., excluding the class triggering the class closure) for different values of R at the time when a class closure is triggered for different values of the probability to test a symptomatic student at school. The boxplot indicates quantile 0.025, 0.25, 0.5, 0.75, and 0.975. The same definition of the boxplot is used throughout the manuscript. Parameters are as the baseline and explored values reported in Supplementary Tab. 1. Note also that *R* is estimated in the absence of the class-closure strategy and the scenario considered is F50. **B** As A, but for the probability to test a symptomatic (non-student) individual in the community. **C** As A, but for the time from symptom onset to sample collection. **D** As A, but for the time from sample collection to laboratory diagnosis.

## 2.2 Daily number of imported infections

In the baseline analysis, we consider that, on average, 1.34 infections are imported per day. To explore the robustness of the results to this choice, we varied the mean daily number of imported infections by assuming two scenarios where the daily number of imported infections is 0.25 and 4 times that of the baseline (i.e., 0.34 and 5.36, respectively). We simulate the impact of the reactive class-closure strategy and found very consistent estimates of the relative change in COVID-19 burden in these 3 scenarios (Supplementary Fig. 5).

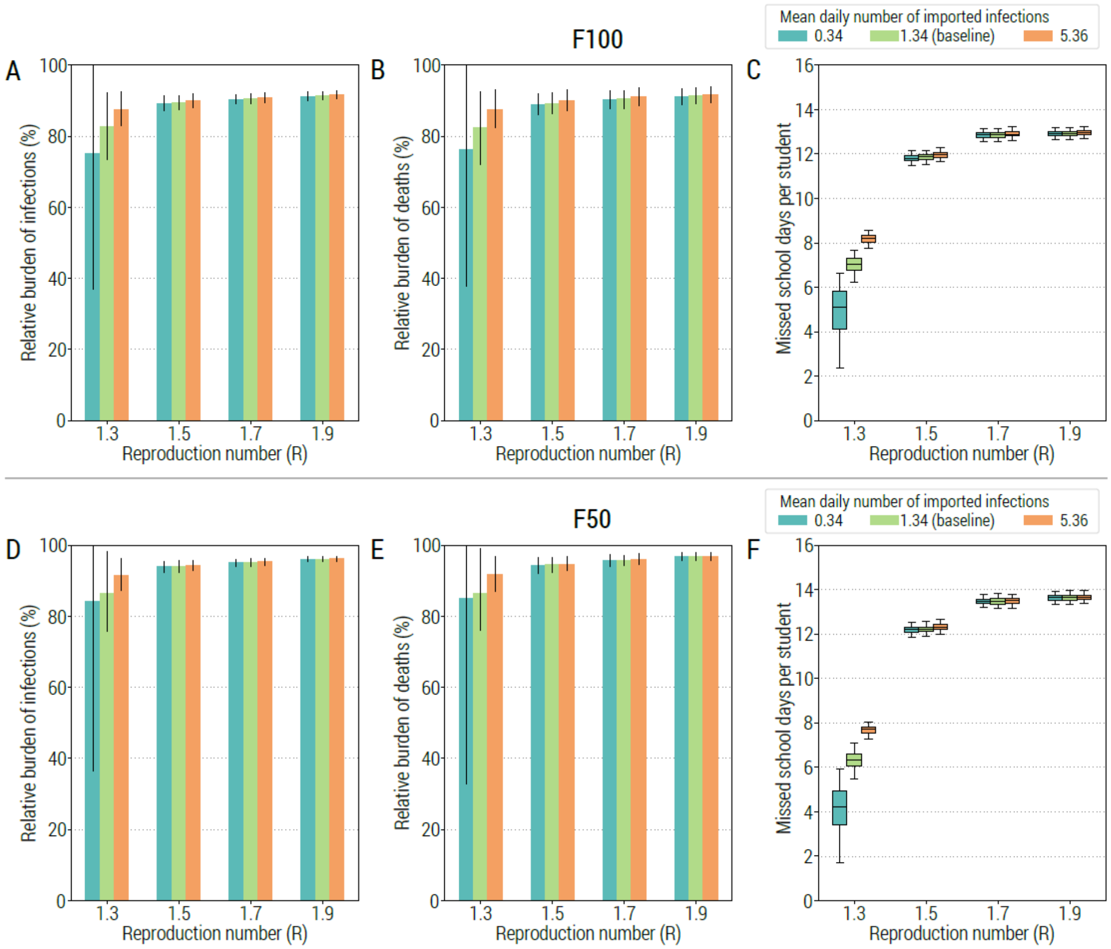


**Supplementary Fig. 5. Sensitivity of the class-closure strategy based on syndromic surveillance to changes in the mean daily imported infectious individuals. A** Relative burden of the cumulative number of infections after one year as a function of the reproduction number and for different initial number of seeds. The bars correspond to the mean value, while the vertical lines represent 95% quantile intervals; colors refer to the baseline value (i.e., 1.34) and two alternative values (i.e., 0.34 and 5.36). Note that *R* is estimated in the absence of the class-closure strategy and the scenario considered is F100. **B** As A, but for the death. **C** Number of missed school days per student. The boxplot indicates quantile 0.025, 0.25, 0.5, 0.75, and 0.975. **D** As A, but for scenario F50. **E** As B, but for scenario F50. **F** As C, but for scenario F50.

## 2.3 Incubation period

In the main analysis, we consider a gamma distributed incubation period with a mean of 6.3 days (sd: 4.3) [3]. Here we conducted a sensitivity analysis where one alternative mean duration of the incubation period, namely 5.2 days (sd: 4.3) was considered [6]. The obtained results are very consistent with those obtained in the main analysis (Supplementary Fig. 6).

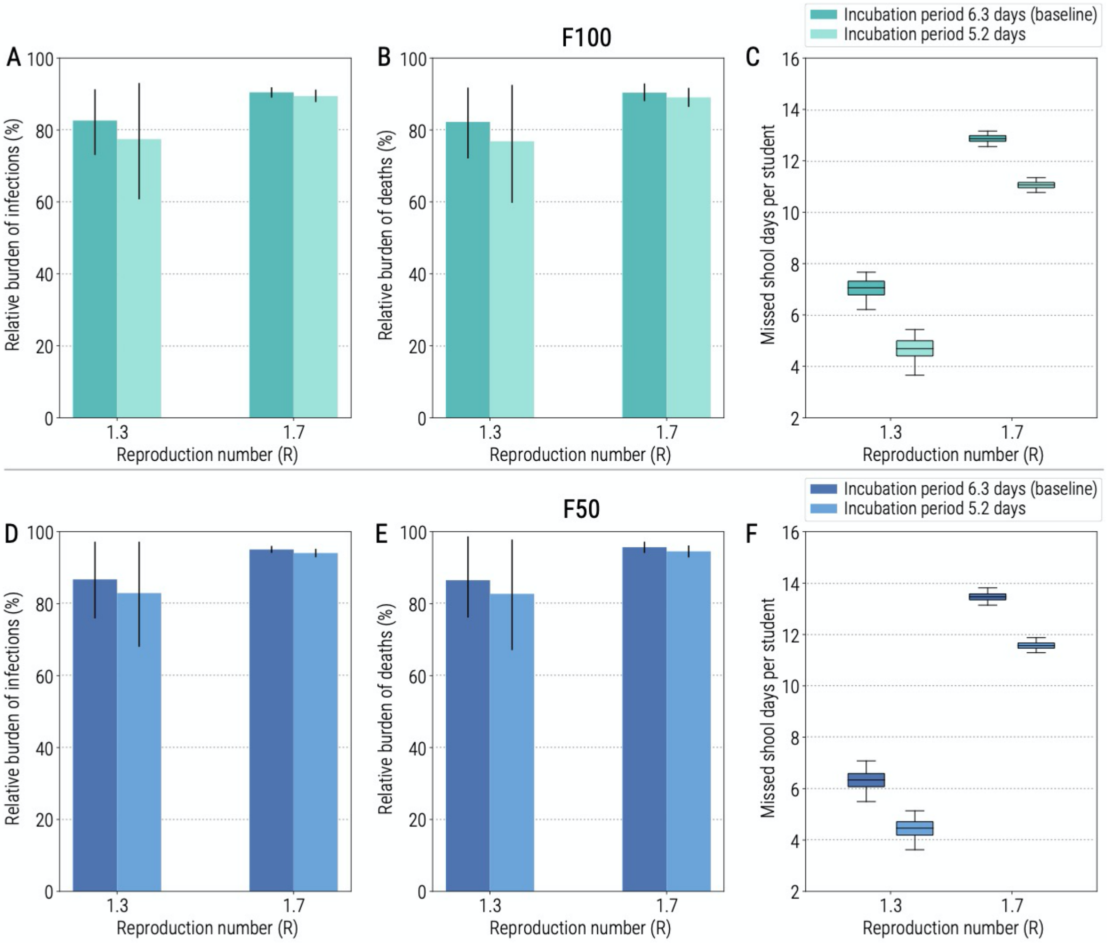


**Supplementary Fig. 6. Sensitivity of the class-closure strategy based on syndromic surveillance to changes in the duration of the incubation period. A** Relative burden of the cumulative number of infections after one year as a function of the reproduction number and for two scenarios about incubation period. The bars correspond to the mean value, while the vertical lines represent 95% quantile intervals; colors refer to the two tested values of the incubation period (namely, 6.3 and 5.2 days). Note that *R* is estimated in the absence of the class-closure strategy and the scenario considered is F100. **B** As A, but for the cumulative number of deaths. **C** Number of missed school days per student due to the reactive class-closure strategy. The boxplot indicates quantile 0.025, 0.25, 0.5, 0.75, and 0.975. **D** As A, but for scenario F50. **E** As B, but for scenario F50. **F** As C, but for scenario F50.

## 2.4 Homogeneous susceptibility to infection by age

In the main analysis, we considered age-specific susceptibility to infection (as estimated in the literature [12]). Here we proposed additional sensitivity analyses where we assume a homogenous susceptibility to infection by age (i.e., $\delta_a$=1 for all ages). All the obtained results are very consistent with those obtained in the main analysis (Supplementary Fig. 7).

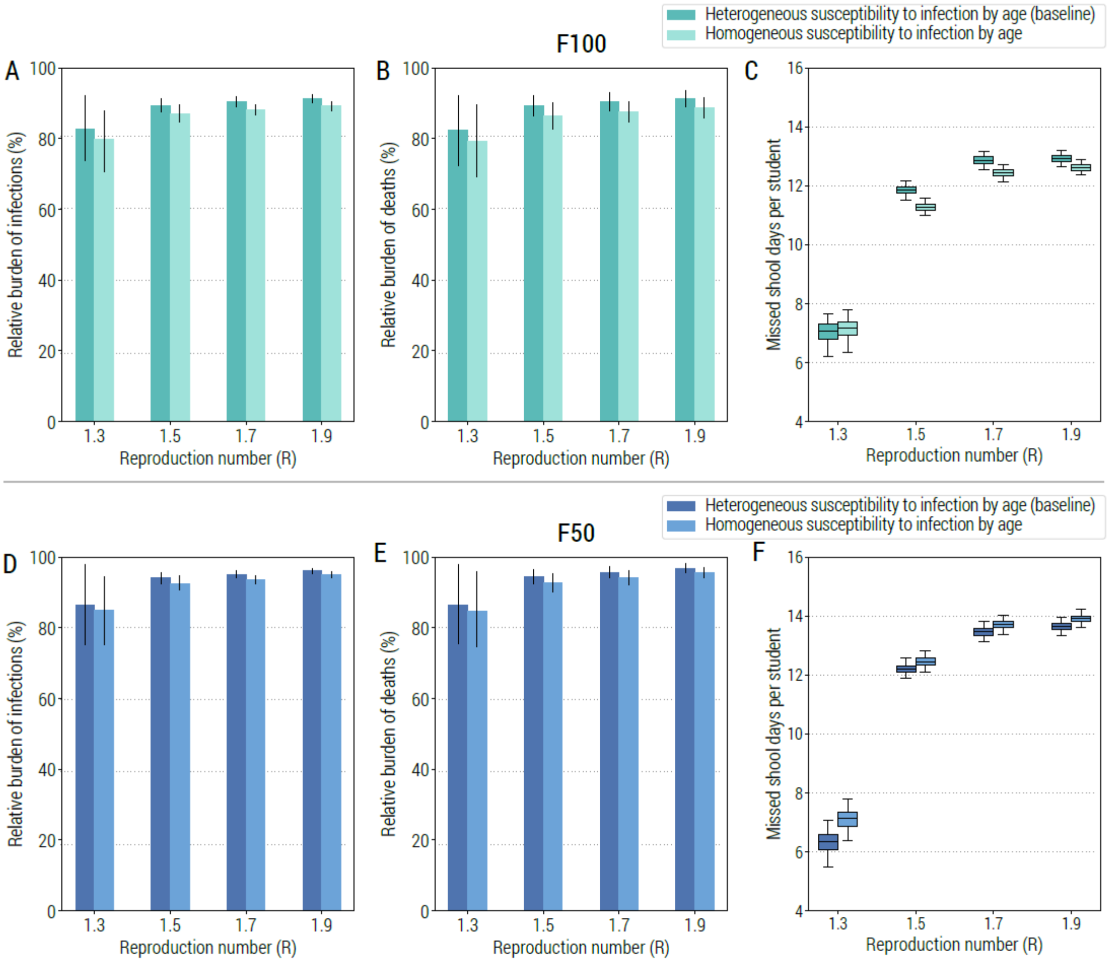


**Supplementary Fig. 7. Sensitivity of the class-closure strategy based on syndromic surveillance to changes in susceptibility to infection by age. A** Relative burden of the cumulative number of infections after one year as a function of the reproduction number and for two scenarios about susceptibility to infection. The bars correspond to the mean value, while the vertical lines represent 95% quantile intervals; colors refer to the age-specific susceptibility (i.e., baseline) and homogeneous susceptibility to infection by age. Note that $R$ is estimated in the absence of the class-closure strategy and the scenario considered is F100. **B** As A, but for the number of deaths. **C** Number of missed school days per student due to the reactive class-closure strategy. The boxplot indicates quantile 0.025, 0.25, 0.5, 0.75, and 0.975. **D** As A, but for scenario F50. **E** As B, but for scenario F50. **F** As C, but for scenario F50.

## 2.5 Infectiousness of asymptomatic vs. symptomatic individuals

In the main analysis, we considered the infectiousness of asymptomatic infected individuals relative to symptomatic one to be equal to 1 (as estimated in Hu et al. [3]). Here we proposed a sensitivity analysis on the infectiousness, where we assume that symptomatic infected individuals transmit twice or four times asymptomatic ones. All the obtained results are very consistent with those obtained in the main analysis (Supplementary Fig. 8).

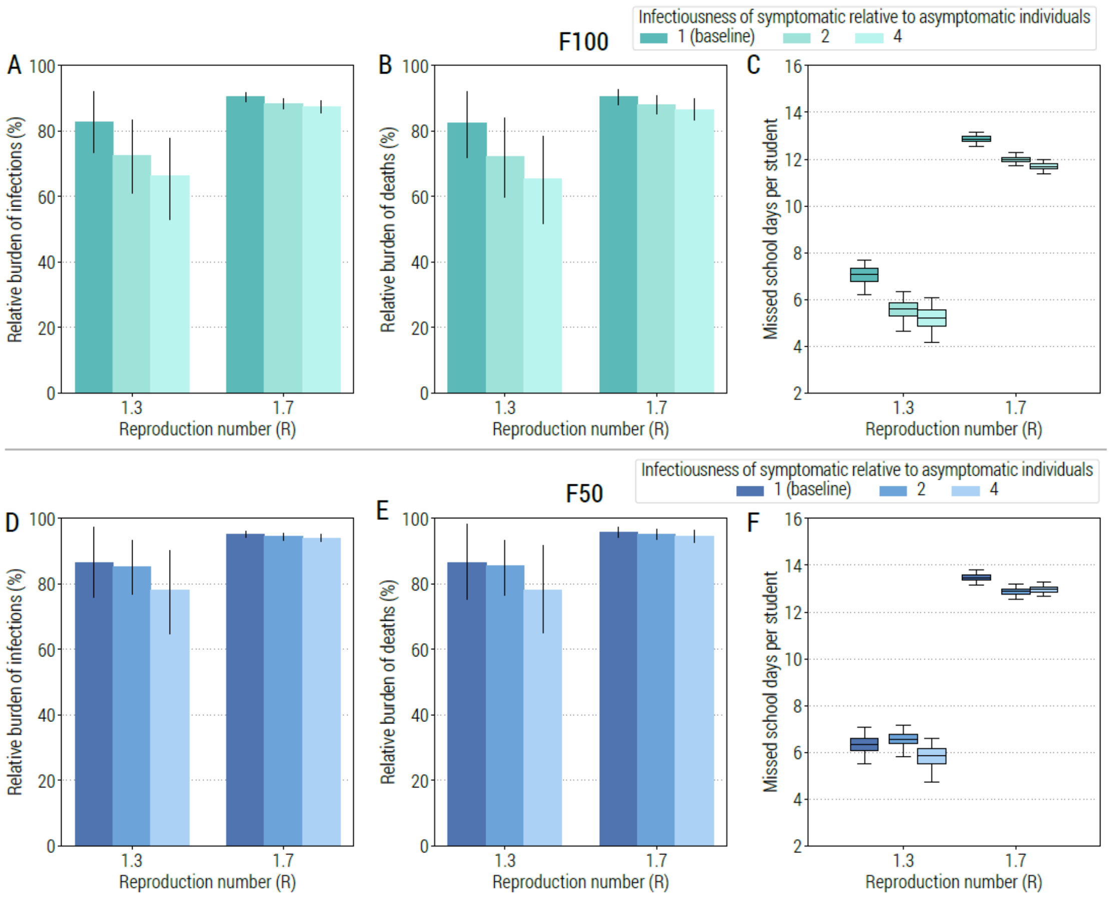


**Supplementary Fig. 8. Sensitivity of the class-closure strategy based on syndromic surveillance to changes in infectiousness of asymptomatic vs. symptomatic individuals. A** Relative burden of the cumulative number of infections after one year as a function of the reproduction number and for two scenarios about relative infectiousness. The bars correspond to the mean value, while the vertical lines represent 95% quantile intervals; colors refer to three scenarios of the infectiousness of symptomatic relative to asymptomatic individuals. Note that $R$ is estimated in the absence of the class-closure strategy and the scenario considered is F100. **B** As A, but for the number of deaths. **C** Number of missed school days per student due to the reactive class-closure strategy. The boxplot indicates quantile 0.025, 0.25, 0.5, 0.75, and 0.975. **D** As A, but for scenario F50. **E** As B, but for scenario F50. **F** As C, but for scenario F50.

## 2.6 Initial fraction of immune population

In the baseline analysis, we consider 5% of the population to be immune at the beginning of the simulation, according to modeling estimates for the Italian population in September 2020 [19]. We performed a sensitivity analysis on the initial immunity of the population by increasing this fraction to 10% and 20%. The obtained results show that the relative change with respect to the simulations without school closure is not affected much by the initial fraction of immune population (Supplementary Fig. 9 A, B, D, and E). However, with the infection attack rate decreasing as the initial fraction of immune population increases, the number of missed school days due to the class-closure strategy remarkably decreases (Supplementary Fig. 9 C and F).

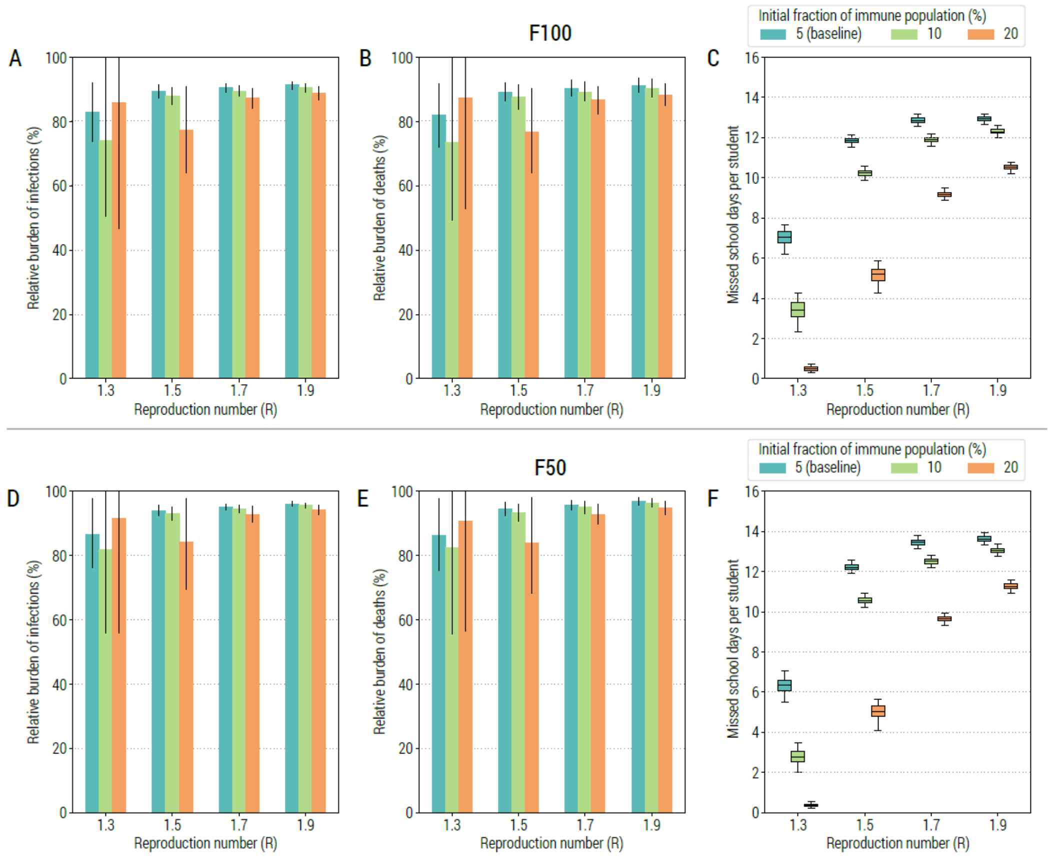


**Supplementary Fig. 9. Sensitivity of the class-closure strategy based on syndromic surveillance to changes in initial fraction of immune population**. **A** Relative burden of the cumulative number of infections after one year as a function of the reproduction number and for three scenarios about initial fraction of immune population. The bars correspond to the mean value, while the vertical lines represent 95% quantile intervals; colors refer to three assumptions of the initial fraction of immune population, including 5% (i.e., the baseline), 10%, and 20%. Note that $R$ is estimated in the absence of the class-closure strategy and the scenario considered is F100. **B** As A, but for the number of deaths. **C** Number of missed school days per student due to the reactive class-closure strategy. The boxplot indicates quantile 0.025, 0.25, 0.5, 0.75, and 0.975. **D** As A, but for scenario F50. **E** As B, but for scenario F50. **F** As C, but for scenario F50.

In addition, we performed another sensitivity analysis assuming an age-dependent initial fraction of immune population. In particular, we considered that 11.2% of individuals aged 18 years or less are immune and 22.4% of the rest of the population is immune (so that the total fraction of immune population is 20%). The obtained results show that the relative burden with respect to the simulations without school closure is not affected much by the age-dependent initial fraction of immune population (Supplementary Fig. 10 A, B, D, and E). However, we estimate that the number of missed school days per student in this scenario is slightly larger than that estimated for the baseline (Supplementary Fig. 10 C and F).

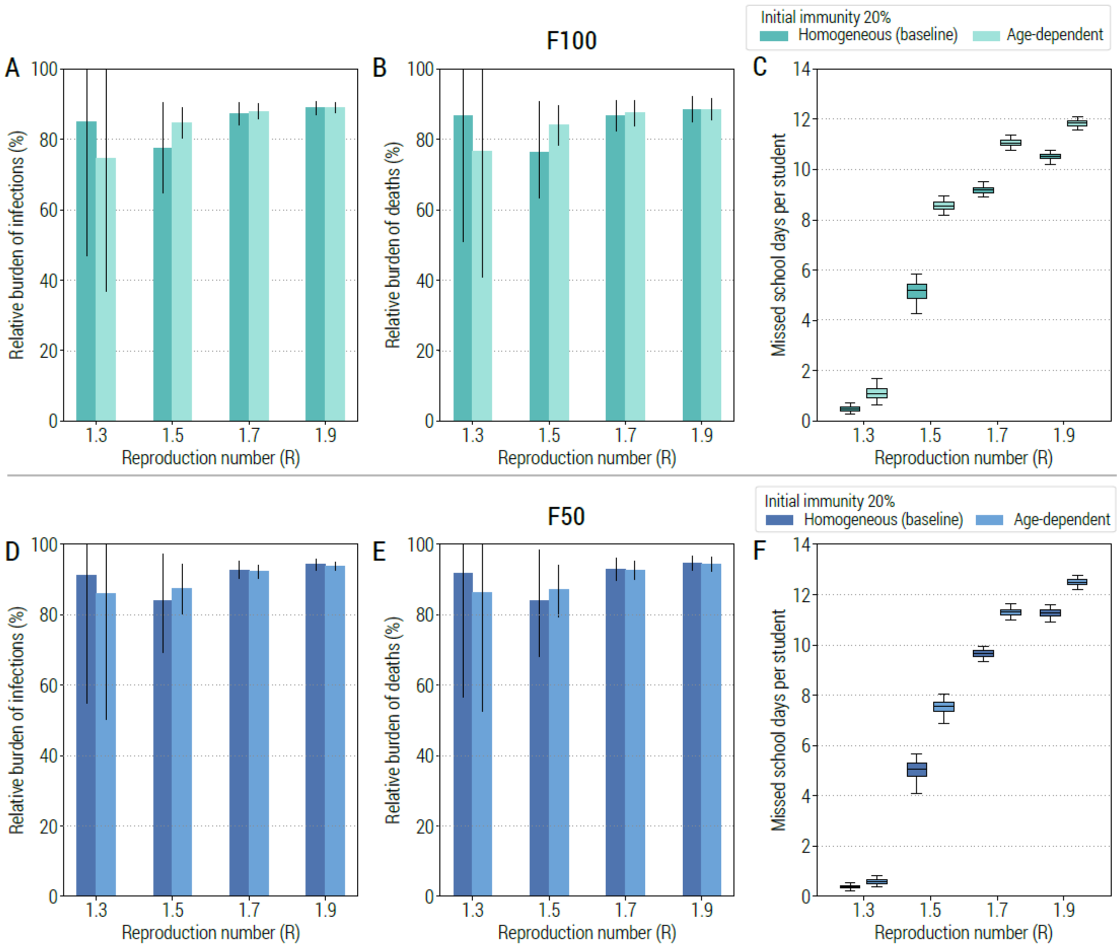


**Supplementary Fig. 10. Sensitivity of the class-closure strategy based on syndromic surveillance to age-dependent initial fraction of immune population**. **A** Relative burden of the cumulative number of infections after one year as a function of the reproduction number and for two scenarios about the age-dependent initial fraction of immunity. The bars correspond to the mean value, while the vertical lines represent 95% quantile intervals; colors refer to two assumptions of the age-dependent initial fraction of immune population, including homogeneous (i.e., the baseline) and heterogeneous across all ages. Note that $R$ is estimated in the absence of the class-closure strategy and the scenario considered is F100. **B** As A, but for the number of deaths. **C** Number of missed school days per student due to the reactive class-closure strategy. The boxplot indicates quantile 0.025, 0.25, 0.5, 0.75, and 0.975. **D** As A, but for scenario F50. **E** As B, but for scenario F50. **F** As C, but for scenario F50.

## 3 Reactive school-closure strategy based on syndromic surveillance

We implemented and tested a reactive school-closure strategy that mirrors exactly the reactive class closure strategy used in the main analysis; the only difference is that once a closure is triggered, the entire school is closed (instead of the single class where a PCR positive student is confirmed).

Supplementary Fig. 11 shows the impact of reactive school-closure policy on the COVID-19 burden and the number of missed school days per student due to the strategy. In the baseline analysis, 10% of initially immune population is used. As compared to the class-closure strategy (Fig. 1 of the main text), the school-closure strategy leads to a remarkably higher reduction of COVID-19 burden (Supplementary Fig. 11 A and B). However, this strategy entails more than 80 missed school days per student (i.e., half of the school year) in most cases (Supplementary Fig. 11C).

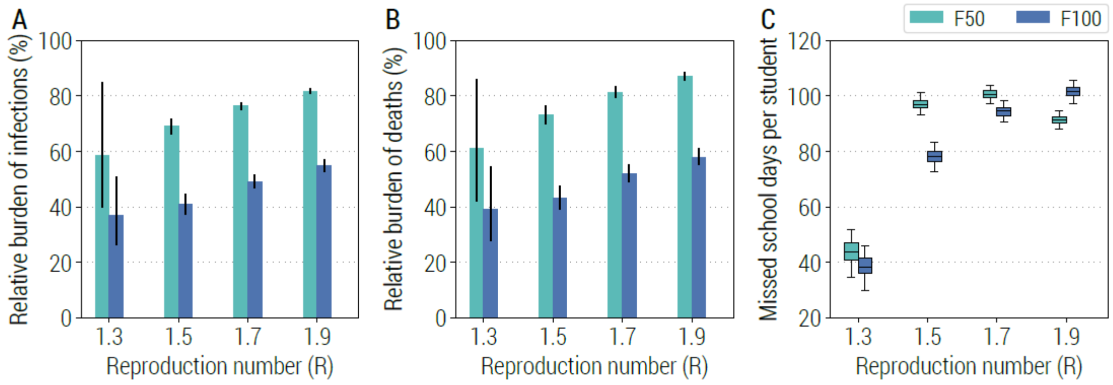


**Supplementary Fig. 11. Impact of the reactive school-closure strategy based on syndromic surveillance.** **A** Relative burden of the cumulative number of infections after one year as a function of the reproduction number and for different scenarios about school transmission contribution. The bars correspond to the mean value, while the vertical lines represent 95% quantile intervals; colors refer to the two scenarios F50 and F100. Parameters are as the baseline values reported in Supplementary Table 1 and 2. Note that $R$ is estimated in the absence of the class-closure strategy. The relative burden is defined as the estimated number of infections after 1 year since the introduction of the first infected individual with the school-closure strategy implemented, relative to the estimated number without the implementation of the school-closure strategy. **B** As A, but for the number of deaths. **C** Number of missed school days per student due to the reactive school-closure strategy. The boxplot indicates quantile 0.025, 0.25, 0.5, 0.75, and 0.975.

We performed a sensitivity analysis on the initial immunity of the population by increasing the fraction of immune population to 15% and 20%. Supplementary Fig. 12 shows the impact of increased fraction of immune population on the COVID-19 burden and the number of missed school days per student due to the reactive school-closure strategy. As observed for the reactive class-closure strategy (Supplementary Fig. 9), the reduction of COVID-19 burden is not much affected by the initial fraction of immune population (Supplementary Fig. 12 A and B). However, the number of missed school days increases

with increases in immunity for low values of $R$, while it decreases for $R$=1.7 and 1.9 (Supplementary Fig. 12 C).

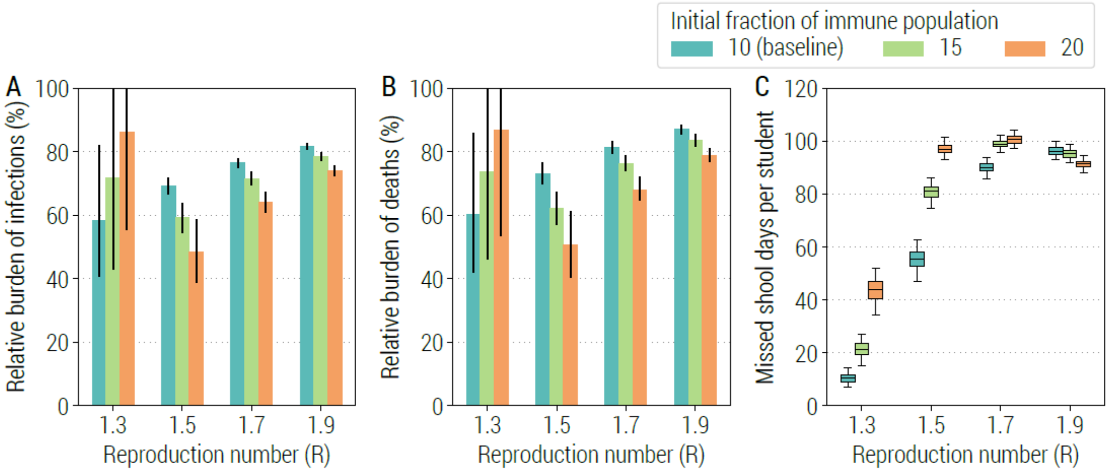


**Supplementary Fig. 12. Sensitivity of the school-closure strategy based on syndromic surveillance to changes in initial fraction of immune population**. **A** Relative burden of the cumulative number of infections after one year as a function of the reproduction number and for three different initial fractions of immune population. The bars correspond to the mean value, while the vertical lines represent 95% quantile intervals; colors refer to three assumptions of the initial fraction of immune population, including 10% (i.e., baseline), 15%, and 20%. Note that $R$ is estimated in the absence of the class-closure strategy and the scenario considered is F50. **B** As A, but for the number of deaths. **C** Number of missed school days per student due to the reactive school-closure strategy. The boxplot indicates quantile 0.025, 0.25, 0.5, 0.75, and 0.975.

# 4 Reactive class-closure strategy based on rapid antigen screening

## 4.1 Additional results for the baseline analysis

In addition to the primary results of COVID-19-related burden shown in Fig. 4A of the main text, Supplementary Fig. 13 shows more metrics related to the burden of COVID-19, including number of symptomatic infections, number of hospitalized patients, number of critical patients, and number of deaths. The results highlight that antigen-based class-closure strategy is capable to prevent not only a large share of infections, but also a significant share of hospitalizations, critical cases, and deaths (Supplementary Fig. 13). In fact, this strategy allows a timely identification of infectious individuals as shown by the low fraction of infectious students in each open class of school (outside the class that triggers the closure) that are infectious at the time of class closure (Supplementary Fig. 14).

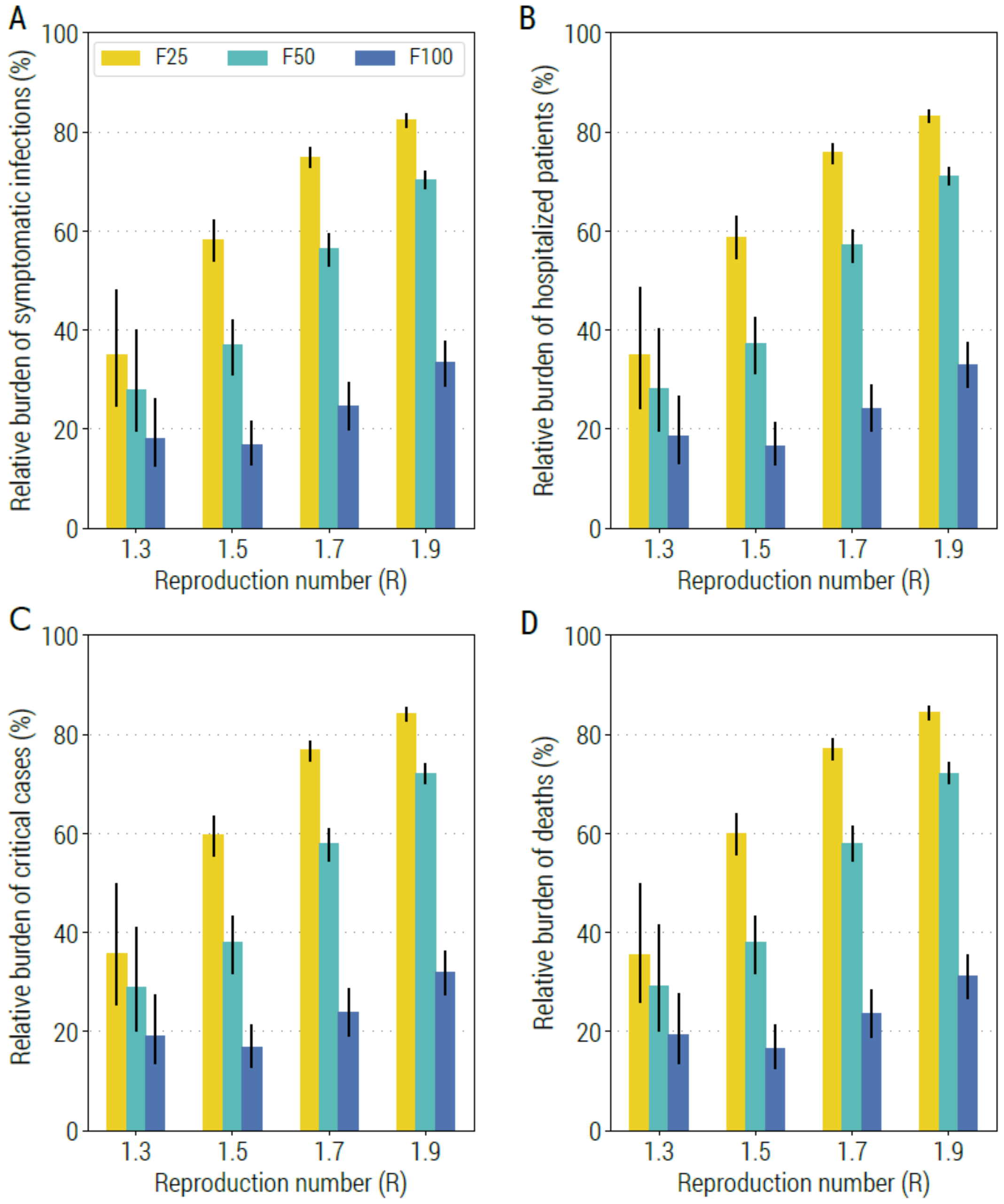


**Supplementary Fig. 13**. **Impact of the reactive class-closure policy based on antigen screening**. **A** Relative burden of the cumulative number of symptomatic infections after one year as a function of the reproduction number and for different scenarios about school transmission contribution. The bar corresponds to the mean value, while the vertical line represents the 95% quantile intervals; colors refer to the three scenarios F25, F50, F100. The fraction of immune population at the beginning of epidemic is set at 10%, the probability of testing a student at school with the antigen test is 100%, the frequency of the antigen screening is weekly; other parameters are as the baseline values reported in Supplementary Table 1 and 2. Note that *R* is estimated in the absence of the class-closure strategy. **B** As A, but for the number of hospitalized patients. **C** As A, but for the number of critical cases. **D** As A, but for the number of deaths.

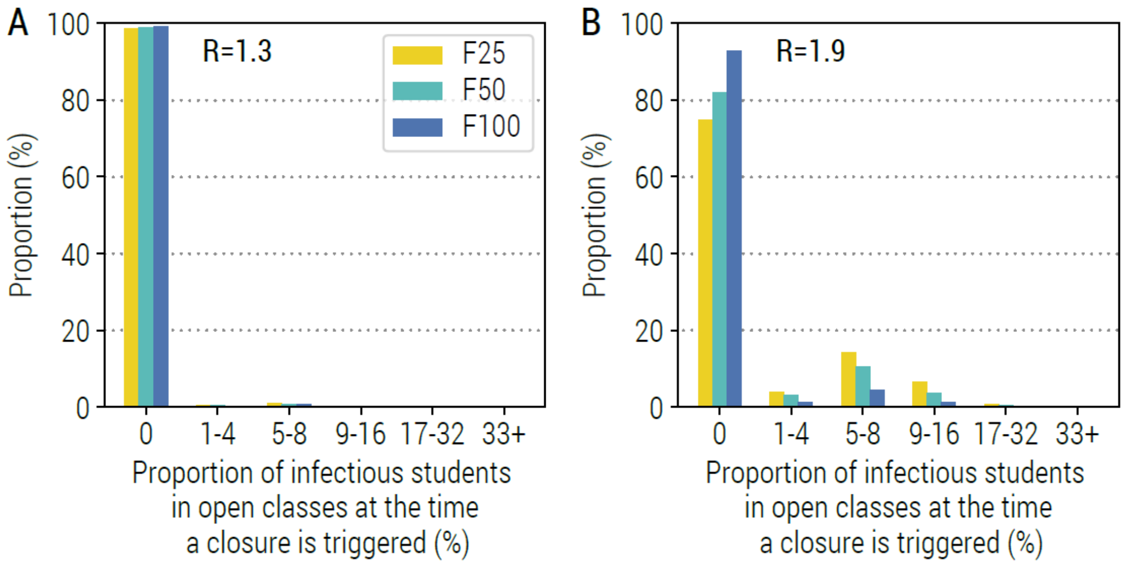


**Supplementary Fig. 14. Infectious students at the time when a class closure is triggered. A** Distribution of the proportion of infectious students in other open classes when one class of the same school is closed for R=1.3. Parameters are as the baseline values reported in Supplementary Table 1 and 2. Note that *R* is estimated in the absence of the class-closure strategy. **B** As in A, but for R=1.9.

## 4.2 Sensitivity of antigen test

In the main analysis, due to the lack of specific data, we considered the sensitivity of antigen tests being independent of the delay between infection and testing (Sec. 1.4). Here we assume that the sensitivity of the antigen test follows the same temporal trend of sensitivity of the RT-PCR test, although with a lower absolute value (i.e., 0.69). All the obtained results are very consistent with those obtained in the main analysis, although the relative burden is slightly increased when a time-varying sensitivity is considered (Supplementary Fig. 15).

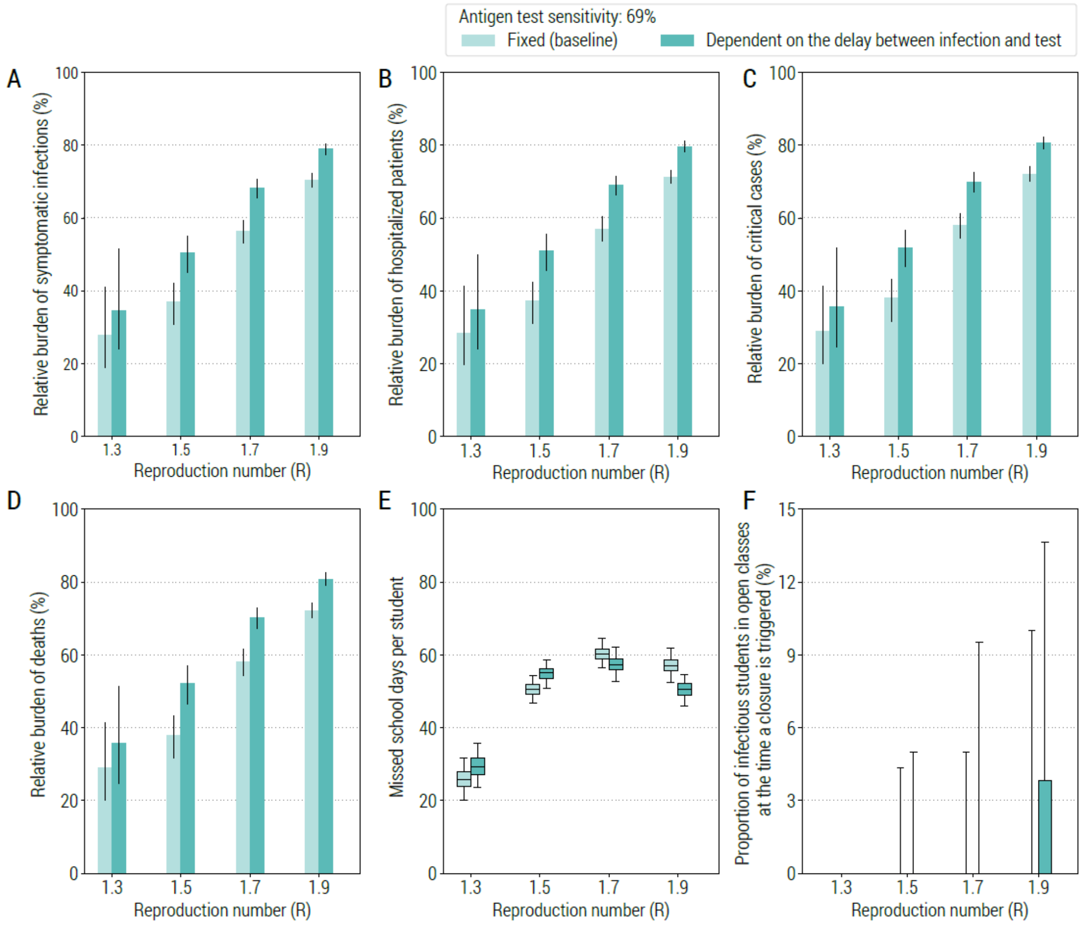


**Supplementary Fig. 15**. **Sensitivity of the class-closure strategy based on antigen screening to the time-varying sensitivity of the antigen test. A** Relative burden of the cumulative number of symptomatic infections after one year as a function of the reproduction number when we use a constant sensitivity of antigen test or a sensitivity that depends on the delay between symptom onset and test. The bars correspond to the mean value, while the vertical lines represent 95% quantile intervals; colors refer to different performance of the strategy. Note that $R$ is estimated in the absence of the class-closure strategy and the scenario considered is F50. **B** As A, but for the number of hospitalized patients. **C** As A, but for the number of critical cases. **D** As A, but for the number of deaths. **E** Number of missed school days per student due to the reactive class-closure strategy. The boxplot indicates quantile 0.025, 0.25, 0.5, 0.75, and 0.975. **F** Proportion of infectious students in open classes at the time when one class of the same school is closed due to the class-closure strategy. The boxplot indicates quantile 0.025, 0.25, 0.5, 0.75, and 0.975.

## 4.3 Antigen screening frequency

In the main text we explored the impact of antigen screening frequency on the effectiveness of the strategy by performing a sensitivity analysis on screening frequency, where we decreased the screening frequency from once every 3 days, to once every 7 days and once every 14 days. Here we show other metrics. Similar to the decrease in the number of infections shown in Fig. 4E (of the main text), the strategy impact on COVID-19 burden (measured through the change in hospitalizations, critical cases, and deaths) tend to decrease with screening becoming less frequent (Supplementary Fig. 16 A-D). The effectiveness of the control over epidemic decreases mostly due to the low number of infectious students at the time the class closure is triggered as compared to strategy based on syndromic surveillance (Supplementary Fig. 16E vs. Fig. 2 of the main text).

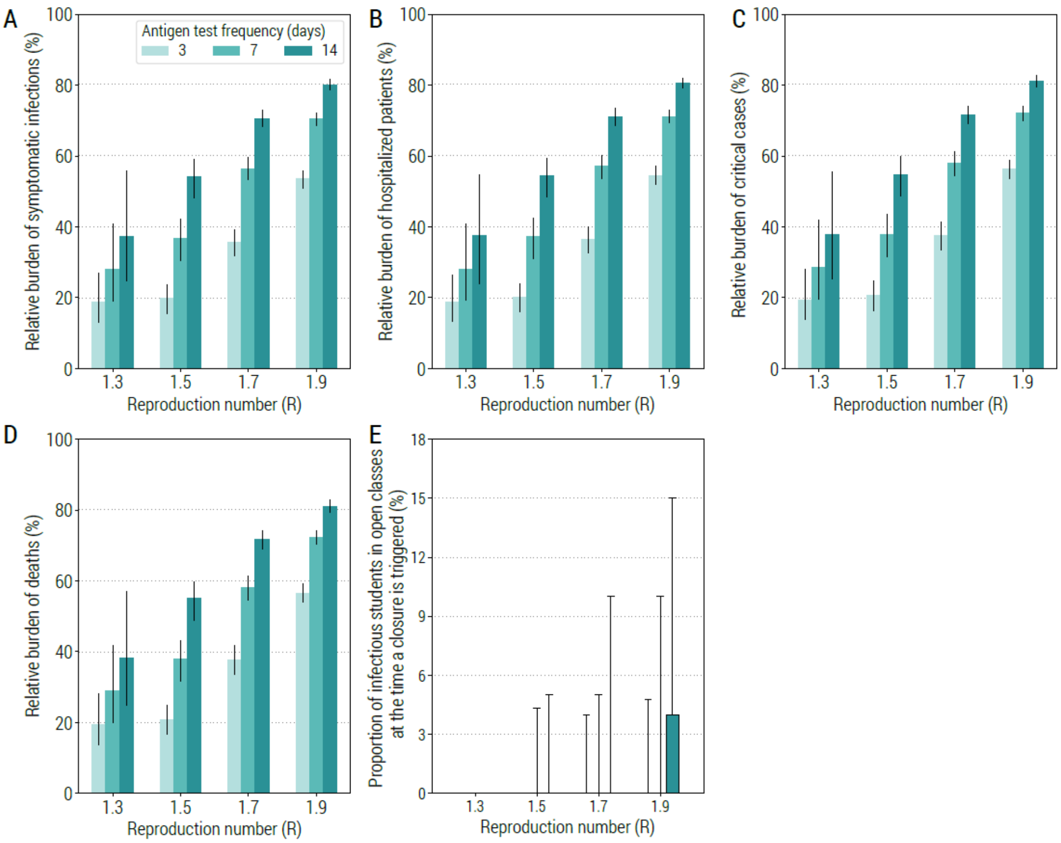


**Supplementary Fig. 16**. **Sensitivity of the class-closure strategy based on antigen screening to changes in screening frequency. A** Relative burden of the cumulative number of symptomatic infections after one year as a function of the reproduction number and for three scenarios about screening frequency (every 3, 7 or 14 days). The bars correspond to the mean value, while the vertical lines represent 95% quantile intervals. Note that $R$ is estimated in the absence of the class-closure strategy and the scenario considered is F50. **B** As A, but for the number of hospitalized patients. **C** As A, but for the number of critical cases. **D** As A, but for the number of deaths. **E** Proportion infectious students in open classes at the time when one class of the same school is closed due to the reactive class-closure strategy. The boxplot indicates quantile 0.025, 0.25, 0.5, 0.75, and 0.975.

In the main text, we considered that the antigen test screening is performed when (at least) one positive individual is identified. Here we consider two alternative scenarios: one when the strategy is always in place (i.e., regardless of whether cases are identified) and another one where the strategy is in place only when 5 (or more) positive individuals are identified. As shown in Supplementary Fig. 17 A-E, the relative burden remarkably decreases when the antigen screening is always in place. However, this entails a much larger cost in terms of missed school days per student (Supplementary Fig. 17F). The proportion of infectious students in open classes at the time of a class closure is lower for all values of $R$ (Supplementary Fig. 17G).

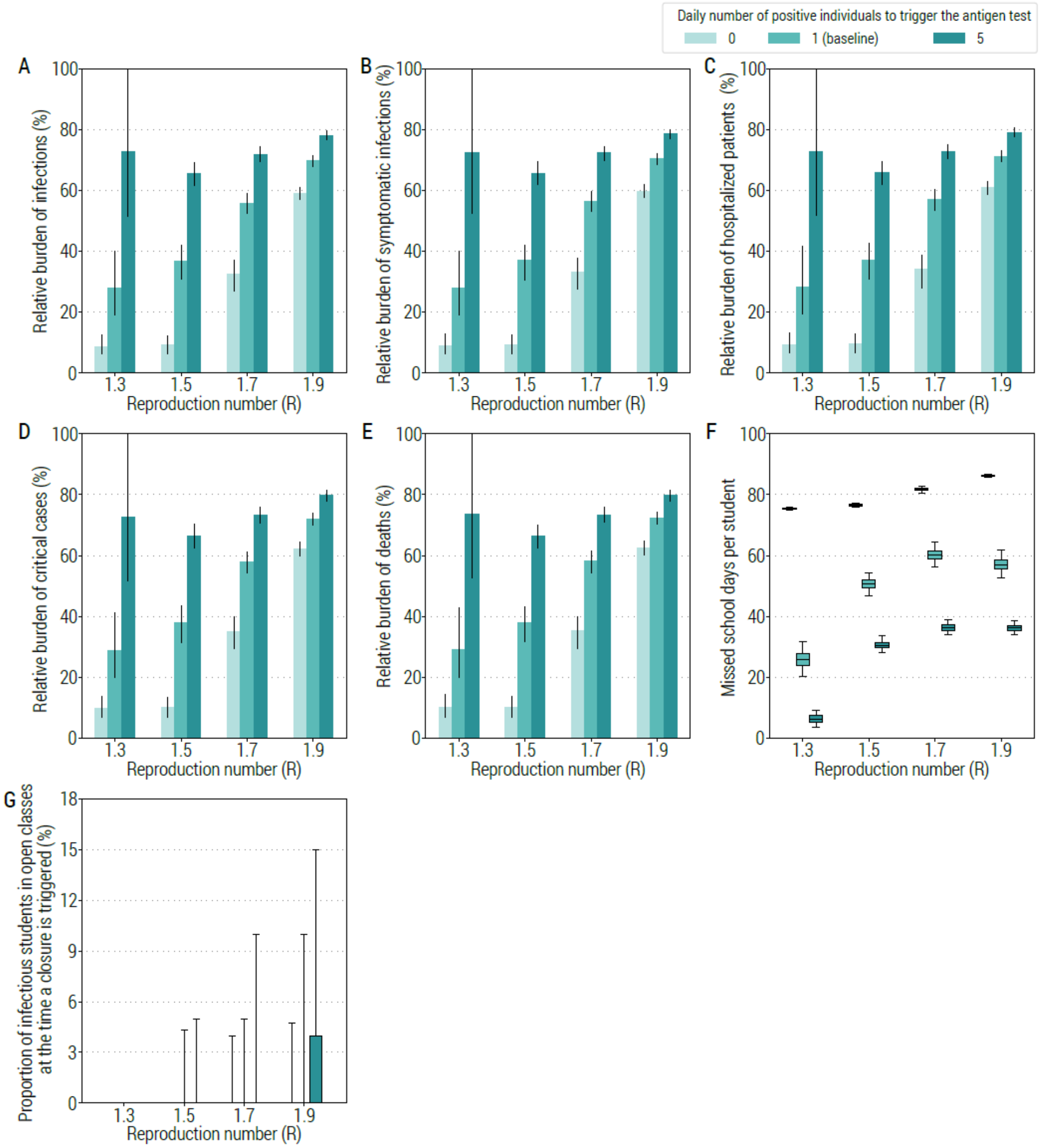


**Supplementary Fig. 17**. **Sensitivity of the class-closure strategy based on antigen screening to the number of positive individuals in the population triggering the antigen screening in schools. A** Relative burden of the cumulative number of infections after one year as a function of the reproduction number when 0, 1+, or 5+ positive individuals trigger the antigen screening. The bars correspond to the mean value, while the vertical lines represent 95% quantile intervals; colors refer to different performance of the strategy. Note that *R* is estimated in the absence of the class-closure strategy and the scenario considered is F50. **B** As A, but for symptomatic infections. **C** As A, but for the number of hospitalized patients. **D** As A, but for the number of critical cases. **E** As A, but for the number of deaths. **F** Number of missed school days per student due to the reactive class-closure strategy. The boxplot indicates quantile 0.025, 0.25, 0.5, 0.75, and 0.975. **G** Proportion of infectious students in open classes at the time when a class of the same school is closed due to the class-closure strategy. The boxplot indicates quantile 0.025, 0.25, 0.5, 0.75, and 0.975.

### 4.4 Initial fraction of immune population

In the analysis of the antigen-based class-closure strategy presented in the main text, we considered 10% of the population to be initially immune. We performed a sensitivity analysis by increasing the fraction of the initially immune population to 15% and 20%. For 20% of initially immune population, the strategy averts 80% of the hospitalizations, critical cases, and deaths for $R$=1.5, and decreases them by almost 70% for $R$=1.7 (Supplementary Fig. 18 A-E). The cost in terms of missed education remarkably decreases (Supplementary Fig. 18 F) and the capacity of the strategy to timely identify infectious students increases (Supplementary Fig. 18 G).

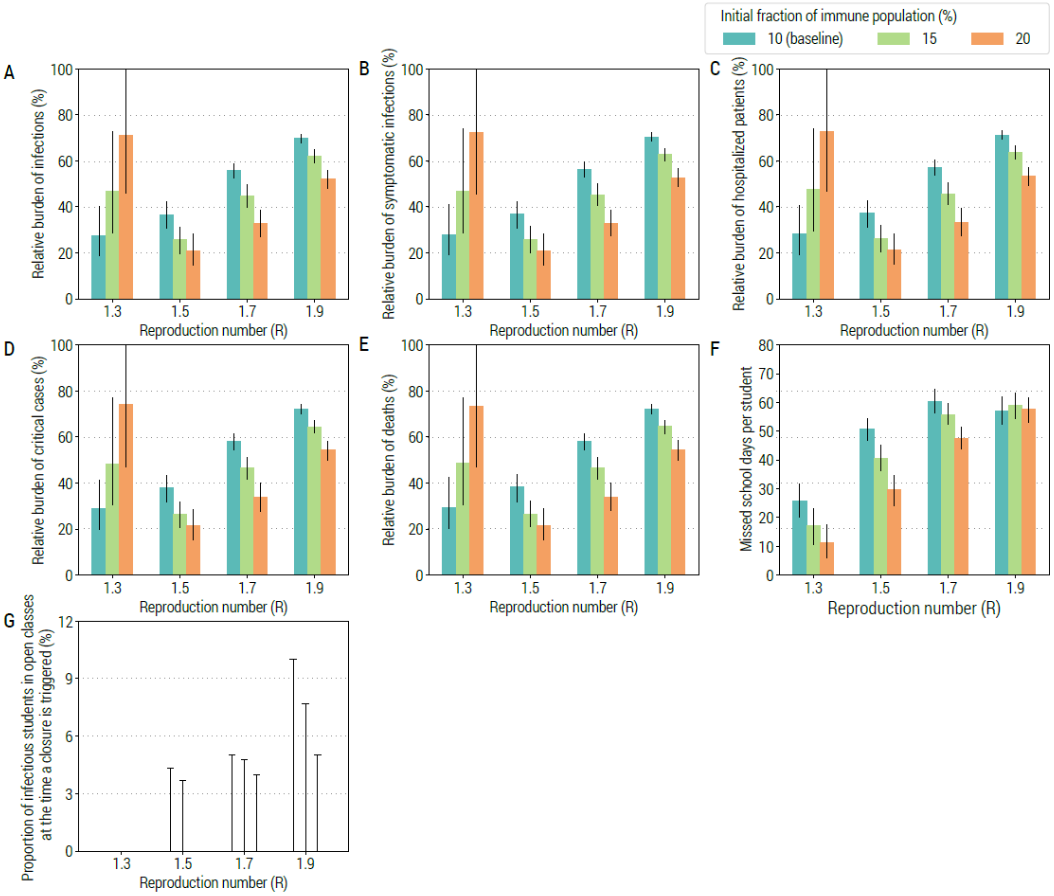


**Supplementary Fig. 18**. **Sensitivity of the class-closure strategy based on antigen screening to changes in initial fraction of immune population**. **A** Relative burden of the cumulative number of infections after one year as a function of the reproduction number and for three different initial fractions of immune population. The bars correspond to the mean value, while the vertical lines represent 95% quantile intervals; colors refer to three assumptions of the initial fraction of immune population, including 10% (i.e., baseline), 15%, and 20%. Note that $R$ is estimated in the absence of the class-closure strategy and the scenario considered is F50. **B** As A, but for symptomatic infections. **C** As A, but for the number of hospitalized patients. **D** As A, but for the number of critical cases. **E** As A, but for the number of deaths. **F** As A, but for number of missed school days per student due to the reactive class-closure strategy. **G** Proportion of infectious students in open classes at the time when a class of the same school is closed due to the class-closure strategy. The boxplot indicates quantile 0.025, 0.25, 0.5, 0.75, and 0.975.

## *Supplementary References*


1. Fumanelli L, Ajelli M, Manfredi P, Vespignani A, Merler S. Inferring the structure of social contacts from demographic data in the analysis of infectious diseases spread. *PLoS Comput Biol* **8**, e1002673 (2012).

2. Statistical Office of the European Commission (Eurostat), Database by themes. Available at http://epp.eurostat.ec.europa.eu (2011).

3. Hu S*, et al.* Infectivity, susceptibility, and risk factors associated with SARS-CoV-2 transmission under intensive contact tracing in Hunan, China. *Nat Commun* **12**, 1533 (2021).

4. He X*, et al.* Temporal dynamics in viral shedding and transmissibility of COVID-19. *Nat Med* **26**, 672-675 (2020).

5. Cereda D*, et al.* The early phase of the COVID-19 outbreak in Lombardy, Italy. *arXiv*, (2020).

6. Zhang J*, et al.* Evolving epidemiology and transmission dynamics of coronavirus disease 2019 outside Hubei province, China: a descriptive and modelling study. *Lancet Infect Dis* **20**, 793-802 (2020).

7. Lavezzo E*, et al.* Suppression of a SARS-CoV-2 outbreak in the Italian municipality of Vo'. *Nature* **584**, 425-429 (2020).

8. EpiCentro, Epidemia COVID-19. Available at https://www.epicentro.iss.it/coronavirus/bollettino/Bollettino-sorveglianza-integrata-COVID-19_20-gennaio-2021.pdf (2021).

9. Marziano V*, et al.* Return to normal: COVID-19 vaccination under mitigation measures. *medRxiv*, 2021.2003.2019.21253893 (2021).

10. Poletti P*, et al.* Association of Age With Likelihood of Developing Symptoms and Critical Disease Among Close Contacts Exposed to Patients with Confirmed SARS-CoV-2 Infection in Italy. *JAMA Network Open* **4**, e211085 (2021).

11. Litvinova M, Liu Q, Kulikov ES, Ajelli M. Reactive school closure weakens the network of social interactions and reduces the spread of influenza. *Proc Natl Acad Sci USA* **116**, 13174 (2019).

12. Viner RM*, et al.* Susceptibility to SARS-CoV-2 Infection Among Children and Adolescents Compared With Adults: A Systematic Review and Meta-analysis. *JAMA Pediatr* **175**, 143-156 (2021).

13. Istituto Superiore di Sanita', Operational guidance for the management of SARS-CoV-2 cases and outbreaks in schools and kindergartens. Available at https://www.iss.it/documents/5430402/0/Rapporto+ISS+COVID+n.+58_Rev+EN.pdf/eb93df69-d22a-cca2-6a8f-8d3c831e2653?t=1604672394638 (2020).

14. Kucirka LM, Lauer SA, Laeyendecker O, Boon D, Lessler J. Variation in False-Negative Rate of Reverse Transcriptase Polymerase Chain Reaction-Based SARS-CoV-2 Tests by Time Since Exposure. *Ann Intern Med* **173**, 262-267 (2020).

15. Istituto Superiore di Sanita', Rapporti ISS COVID-19 - ISS. Available at https://www.iss.it/rapporti-covid-19 (2020).

16. Zardini A, *et al.* A quantitative assessment of epidemiological parameters to model COVID- 19 burden. *arXiv*, 2103.15780 (2021).

17. Poletti P, *et al.* Age-specific SARS-CoV-2 infection fatality ratio and associated risk factors, Italy, February to April 2020. *Euro Surveill* **25**, 2001383 (2020).

18. Wallinga J, Lipsitch M. How generation intervals shape the relationship between growth rates and reproductive numbers. *Proc Biol Sci* **274**, 599-604 (2007).

19. Marziano V, *et al.* Retrospective analysis of the Italian exit strategy from COVID-19 lockdown. *Proc Natl Acad Sci USA* **118**, e2019617118 (2021).

20. National Center for Immunization and Respiratory Diseases (NCIRD), Interim Guidance for Antigen Testing for SARS-CoV-2. Available at https://www.cdc.gov/coronavirus/2019-ncov/lab/resources/antigen-tests-guidelines.html (2020).

21. Chaimayo C, *et al.* Rapid SARS-CoV-2 antigen detection assay in comparison with real-time RT-PCR assay for laboratory diagnosis of COVID-19 in Thailand. *Virol J* **17**, 177 (2020).

22. Diao B, *et al.* Accuracy of a nucleocapsid protein antigen rapid test in the diagnosis of SARS-CoV-2 infection. *Clin Microbiol Infect* **27**, 289 e281-289 e284 (2021).

23. Lambert-Niclot S, *et al.* Evaluation of a Rapid Diagnostic Assay for Detection of SARS-CoV-2 Antigen in Nasopharyngeal Swabs. *J Clin Microbiol* **58**, (2020).

24. Mertens P, *et al.* Development and Potential Usefulness of the COVID-19 Ag Respi-Strip Diagnostic Assay in a Pandemic Context. *Front Med (Lausanne)* **7**, 225 (2020).

25. Nalumansi A, *et al.* Field Evaluation of the Performance of a SARS-CoV-2 Antigen Rapid Diagnostic Test in Uganda using Nasopharyngeal Samples. *Int J Infect Dis* **104**, 282-286 (2020).

26. Porte L*, et al.* Evaluation of a novel antigen-based rapid detection test for the diagnosis of SARS-CoV-2 in respiratory samples. *Int J Infect Dis* **99**, 328-333 (2020).

27. Weitzel T*, et al.* Comparative evaluation of four rapid SARS-CoV-2 antigen detection tests using universal transport medium. *Travel Med Infect Dis* **39**, 101942 (2020).
